# Scaling Up Q-Learning via Exploiting State–Action Equivalence

**DOI:** 10.3390/e25040584

**Published:** 2023-03-29

**Authors:** Yunlian Lyu, Aymeric Côme, Yijie Zhang, Mohammad Sadegh Talebi

**Affiliations:** 1 Department of Computer Science, University of Copenhagen, Universitetsparken 1, 2100 Copenhagen, Denmark; yizh@di.ku.dk (Y.Z.); m.shahi@di.ku.dk (M.S.T.); 2 School of Computer Science and Engineering, University of Electronic Science and Technology of China, Xiyuan Ave., Chengdu 611731, China; 3 Inria Rennes, Bretagne Atlantique Campus Universitaire de Beaulieu, Avenue du Général Leclerc, 35042 Rennes, France; aymeric.come@inria.fr

**Keywords:** reinforcement learning, Markov decision process, Q-learning, equivalence structure

## Abstract

Recent success stories in reinforcement learning have demonstrated that leveraging structural properties of the underlying environment is key in devising viable methods capable of solving complex tasks. We study off-policy learning in discounted reinforcement learning, where some equivalence relation in the environment exists. We introduce a new model-free algorithm, called QL-ES (Q-learning with equivalence structure), which is a variant of (asynchronous) Q-learning tailored to exploit the equivalence structure in the MDP. We report a non-asymptotic PAC-type sample complexity bound for QL-ES, thereby establishing its sample efficiency. This bound also allows us to quantify the superiority of QL-ES over Q-learning analytically, which shows that the theoretical gain in some domains can be massive. We report extensive numerical experiments demonstrating that QL-ES converges significantly faster than (structure-oblivious) Q-learning empirically. They imply that the empirical performance gain obtained by exploiting the equivalence structure could be massive, even in simple domains. To the best of our knowledge, QL-ES is the first provably efficient model-free algorithm to exploit the equivalence structure in finite MDPs.

## 1. Introduction

Reinforcement learning (RL) aims to develop computer systems with the ability to learn how to behave optimally, or nearly so, in an unknown dynamic environment. An RL task typically involves an agent interacting with the environment, which is often modeled as a Markov decision process (MDP), and the agent’s goal is to find a policy maximizing some notion of reward. In most settings in RL, the MDP is initially unknown beyond its state and action spaces. Hence, the agent aims to learn a near-optimal policy using the experiences collected from the environment.

A classical setting in RL is off-policy learning [1], where one tries to learn the optimal action–value function (i.e., Q-function) through the data collected under some *behavior* or *logging* policy. Perhaps the most famous off-policy learning algorithm is the celebrated Q-learning algorithm [2], whose improved variants, combined with deep neural networks as function approximators, played key roles in many recent breakthroughs in RL [3,4]. More precisely, Q-learning and its variants fall under the category of *model-free* methods, in which one tries to directly estimate the optimal value function—without estimating the true model (MDP)—from the collected experience, from which a near-optimal policy could be straightforwardly derived. This approach is in stark contrast to the *model-based* counterpart, where one first attempts to estimate the unknown model parameters (i.e., MDP parameters that include transition probabilities and rewards) from the collected experience and then finds an optimal policy in the estimated model.

Off-policy learning in finite MDPs is by now well understood, and the existing literature (e.g., [5,6,7,8,9]) exhibit algorithms that admit PAC-type sample complexity guarantees. Precisely speaking, these algorithms are guaranteed to return a near-optimal policy, with respect to a prescribed accuracy, with high probability if the amount of collected experience exceeds a certain (algorithm-dependent) function of the MDP parameters (and relevant input parameters). Most of these works study unstructured tabular MDPs, where the advertised sample complexity bounds scale, among other things, with the size of the state–action space. Thus, despite their appealing performance guarantees, most of these algorithms only work reasonably well when the size of the underlying MDP is small. On the other hand, many practical tasks can be modeled by MDPs with huge state spaces (or even infinite), but they often exhibit some structural properties. Ignoring such structural properties and directly applying the above algorithms would lead to a prohibitively large sample complexity bound, which may imply a huge learning phase in the worst case. Alternatively, one could leverage the structure in the MDP to speed up the exploration. In fact, exploiting the structure allows the agent to use the collected observations from the environment to reduce the uncertainty in the model parameter for many *similar* state–action pairs at each time slot. As a result, the learning performance would depend on the effective size of the state space (or the effective number of unknown parameters). Various notions of structures have been studied in MDPs, which include the Lipschitz continuity of MDP parameters (e.g., rewards and transition functions) [10,11,12,13], factorization structure [14,15,16], and equivalence relations [17,18,19,20,21,22]. These works reveal that exploiting the underlying structure in the environment in various RL tasks leads to massive empirical performance gain (over structure-oblivious algorithms) and to significantly improved performance bounds. However, exploiting structure often poses additional challenges.

This work is motivated by tabular RL problems, where the (potentially large) state–action space admits a natural partitioning such that within each element of the partition (or class), the state–action pairs have *similar* transition probabilities. There exist several ways to characterize the similarity between the transition distribution of two state–action pairs. Here, we consider a notion implying that they are (almost) identical up to some permutation. As we shall see in later sections, this notion of structure induces an equivalence relation in the state–action space. This model has been considered in prior work [18,23,24], where model-based algorithms were presented to exploit such a structure in the context of regret minimization in episodic or average-reward MDPs. However, their proposed ideas and techniques are specific to a model-based approach, where the model parameters are directly estimated, and cannot be used to incorporate the knowledge of the equivalence structure into a model-free algorithm. Model-free algorithms have played a pivotal role in the recent success of RL to solving complex tasks arising in real-world applications (e.g., autonomous driving and continuous control [25]). Hence, it sounds promising to study the gain one could obtain using model-free methods when leveraging the equivalence structure, thereby extending the theoretical analysis in [18,24] beyond model-based methods.

**Contributions.** We make the following contributions. We study off-policy learning in discounted finite MDPs, admitting some equivalence structure in their state–action space. We introduce a new model-free algorithm, called QL-ES (Q-learning with equivalence structure), which is a variant of (asynchronous) Q-learning tailored to exploit the equivalence structure in the MDP, when a prior knowledge on the structure is provided to the agent. We report a non-asymptotic PAC-type sample complexity bound for QL-ES, thereby establishing its sample efficiency. This bound also allows us to quantify the superiority of QL-ES over Q-learning analytically. As it turns out, the sample efficiency gain of QL-ES over Q-learning is captured by an MDP-dependent quantity ξ that is defined in terms of the associated covering times in the MDP; see Section 5 for details. Analytically establishing the dependence of the gain ratio ξ on the number *S* of states in a given MDP seems difficult, although it is possible to numerically compute it. Nonetheless, we present a simple example where ξ=O(S), thus showcasing that QL-ES in some domains may require *much* fewer (by a factor of *S*) samples than Q-learning. Furthermore, we numerically compute ξ for a few families of MDPs built using standard environments (with increasing *S*), thereby showcasing the theoretical superiority of QL-ES over Q-learning. Through extensive numerical experiments on standard domains, we show that Q-function estimates under QL-ES converge much faster than those obtained from (structure-oblivious) Q-learning. These results demonstrate that the empirical performance gain from exploiting the equivalence structure could be massive, even in simple domains. To our best knowledge, QL-ES is the first provably efficient model-free algorithm to exploit the equivalence structure in MDPs.

## 2. Related Work

**Similarity and equivalence in MDPs.** There is a rich literature on learning and exploiting various notions of structure in MDPs, where the aim is to leverage structure to alleviate the computational cost of finding an optimal policy (in the known MDP setting) or to speed up exploration (in the RL setting). Many such algorithms fall into the category of state abstraction (or aggregation) [26,27]. Approximate homomorphism is proposed to construct beneficial abstract models in MDPs [28]. In the known MDP setting, Refs. [29,30] appear to be the first presenting the notion of equivalence between states based on *stochastic bi-simulation*. The authors of [31,32] use *bi-simulation metrics* as quantitative analogues of the equivalence relations to partition the state space by capturing similarities. In the RL setting, Refs. [18,19,20,33,34] investigate *model-based* algorithms that rely on the grouping of similar states (or state–action pairs) to speed up exploration. Ref. [20] is the first to present an average-reward RL algorithm (in the regret setting), where the confidence intervals of similar states are aggregated. Ref. [18] studies regret minimization in average-reward MDPs with equivalence structure and presents the C-UCRL algorithm, which is capable of exploiting the structure. The regret bound for C-UCRL depends on the number of classes in the MDP rather than the size of the state–action space. A similar equivalence structure was studied in [17] in the context of multi-task RL, where similarities of the transition dynamics across tasks were extracted and exploited to speed up learning. Ref. [24] studies the efficiency of hierarchical RL in the regret setting in scenarios where the hierarchical structure is defined with respect to the notion of equivalence; more precisely, it assumes that the underlying MDP can be decomposed into *equivalent* sub-MDPs—i.e., smaller MDPs with identical reward and transition functions up to some known bijection mappings. Closest to our work, in terms of the structure definition, is [18]. However, we restrict ourselves to a model-free approach where the model-based machinery presented in [18] does not apply. Finally, we mention that there is some literature on exploiting equivalence in deep RL (e.g., [21,22]). However, none of these works study provably efficient learning methods to our best knowledge.

**Q-learning and its variants.** We provide a very brief overview of the works studying theoretical analysis of Q-learning and its variants. Q-learning [2] has been around for more than three decades as a cheap and popular model-free method to solve finite, unknown discounted MDPs without estimating the model. Its convergence was investigated in an asymptotic flavor [35,36], and more recently in the non-asymptotic (finite-sample) regime in a series of work, including [9,37,38,39,40]. To the best of our knowledge, Ref. [9] reports the sharpest PAC-type sample complexity bound for the classical Q-learning. Some of these works present variants of Q-learning with improved sample complexity bounds using a variety of techniques, such as acceleration and variance reduction [9,40,41]. Although the concept of equivalence in MDPs is not new, there is no work reporting PAC-type sample complexity bounds for model-free algorithms combined with equivalence relations, to our knowledge.

## 3. Problem Formulation

In this section, we present some necessary background and formulate the reinforcement learning problem considered in this paper. We use the following notations throughout. For a set *B*, Δ(B) denotes the set of all probability distributions over *B*. For an event *E*, IE denotes the indicator function of *E*: namely, it equals 1 if *E* holds, and 0 otherwise.

### 3.1. Discounted Markov Decision Processes

Let M=(S,A,P,R,γ) be an infinite-horizon discounted MDP [42], where S denotes a discrete state space with cardinality *S*, A denotes a discrete action space with cardinality *A*, and γ∈(0,1) is a discount factor. P:S×A→Δ(S) represents the transition function such that $P(s^{\prime }|s,a)$ denotes the probability of transiting to state $s^{\prime }$ when action a∈A is chosen in state s∈S. Further, R:S×A→[0,1] denotes the reward function supported on [0,1] such that R(s,a) denotes the reward distribution when choosing action a∈A in state s∈S. We denote by $\bar{R}\left(s,a\right)$ the mean of R(s,a). A stochastic (or randomized) policy π:S→Δ(A) is a mapping that maps a state to a probability distribution over A. For a policy π, the value function of π is a mapping $V^{\pi }:S\to R$ defined as

$$V^{\pi }\left(s\right):=E\left[\sum_{t=0}^{\infty }\gamma^{t}r_{t}|s_{0}=s\right]\;,\;s\in S,$$

where for all t≥0, $a_{t}∼\pi \left(s_{t}\right)$, $s_{t+1}∼P\left(\cdot |s_{t},a_{t}\right)$, and $r_{t}∼R\left(s_{t},a_{t}\right)$, and where the expectation is taken with respect to the randomness in rewards, next states, and actions sampled from π. The action–value function of a policy π, denoted by $Q^{\pi }:S\times A\to R$, is defined as

$$Q^{\pi }\left(s,a\right):=E\left[\sum_{t=0}^{\infty }\gamma^{t}r_{t}|s_{0}=s,a_{0}=a\right]\;,\;s\in S,a\in A.$$

The optimal value function is denoted by $V^{⋆}$ and satisfies $V^{⋆}\left(s\right)=\max_{\pi }V^{\pi }\left(s\right)$ for all s∈S. It is well known that in any finite MDP, there exists a stationary deterministic policy $\pi^{⋆}:S\to A$ such that $V^{\pi^{⋆}}=V^{⋆}$, which is called an optimal policy [42]. Similarly, the optimal state–action value function is defined as $Q^{⋆}\left(s,a\right)=\max_{\pi }Q^{\pi }\left(s,a\right)$ for all (s,a)∈S×A. An optimal policy $\pi^{⋆}$ satisfies $\pi^{⋆}\left(s\right)\in \arg\max_{a}Q^{⋆}\left(s,a\right)$ for all s∈S. Furthermore, $Q^{⋆}$ is the unique solution to the optimal Bellman equation [42]:

$$Q^{⋆}\left(s,a\right)=\bar{R}\left(s,a\right)+\gamma \sum_{s^{\prime }\in S}P\left(s^{\prime }|s,a\right)\max_{a^{\prime }\in A}Q^{⋆}\left(s^{\prime },a^{\prime }\right)\;,\;s\in S,a\in A.$$

### 3.2. The Off-Policy Learning Problem and Q-Learning

We consider the off-policy learning problem as follows. The agent is provided with some dataset D collected according to some *behavior or logging* policy $\pi_{b}$. Precisely speaking, D takes the form of trajectory ${\left\{\left(s_{t},a_{t},r_{t}\right)\right\}}_{t\geq 0}$, where $s_{0}$ is some initial state, and where for each t≥0, $a_{t}∼\pi_{b}\left(s_{t}\right)$, $s_{t+1}∼P\left(\cdot |s_{t},a_{t}\right)$, and $r_{t}∼R\left(s_{t},a_{t}\right)$. The agent is given an accuracy parameter ε>0 and a failure probability parameter δ∈(0,1), and its goal is to find an ε-optimal policy using as few samples as possible from D.

We need to impose some assumptions on the behavior policy $\pi_{b}$ to ensure that it is possible to learn a near-optimal policy only using D efficiently with PAC-type guarantees. To state the assumptions, we introduce some necessary definitions, which are borrowed from standard textbooks on Markov chains (e.g., [43]) but are also standard in the theoretical analysis of Q-learning (e.g., [9,39]). Let X be a finite set. The *total variation distance* between two distributions μ and ν defined over X is $d_{\mathrm{TV}}\left(\mu ,\nu \right):=\frac{1}{2}\sum_{x\in X}\left|\mu (x)-\nu (x)\right|$. Now, consider an ergodic Markov chain ${\left(X_{t}\right)}_{t\geq 1}$ with state space X and transition function p∈Δ(X), and let μ be the unique stationary distribution of the chain. The Markov chain is said to be *uniformly ergodic* if there exist some ρ<1 and M<∞ such that for all t>0,

$$\underset{x\in X}{\sup}d_{\mathrm{TV}}\left(\mu ,p^{t}\left(\cdot |x\right)\right)\leq M\rho^{t},$$

where $p^{t}\left(\cdot |x\right)$ is the distribution of $X_{t}$ given $X_{0}=x$.

Similar to [9], we assume that the Markov chain induced by $\pi_{b}$ is uniformly ergodic. This property ensures that all the states are visited infinitely often, and that convergence to the stationary distribution is performed at a geometric pace. This property is needed for the result presented in Section 5.

**The Q-learning algorithm.** The Q-learning algorithm [35] is perhaps the most famous model-free algorithm for learning an optimal policy in unknown tabular MDPs. As a model-free method, it directly learns the optimal Q-function $Q^{⋆}$ of the MDP (without estimating *P* and *R*), which can be used to derive a policy. The algorithm maintains an estimate $Q_{t}$ of the optimal $Q^{⋆}$ at each time step *t*. Specifically, it starts from an arbitrary choice for $Q_{0}\in R^{S\times A}$ and updates $Q_{t}$, at each t≥0, as

(1) $$\begin{array}{c} Q_{t+1}\left(s_{t},a_{t}\right)=\left\{\begin{array}{cc} \left(1-\alpha_{t}\right)Q_{t}\left(s,a\right)+\alpha_{t}\left(r_{t}+\gamma \max_{a^{\prime }\in A}Q_{t}\left(s_{t+1},a^{\prime }\right)\right), & \left(s,a\right)=\left(s_{t},a_{t}\right),\\ Q_{t+1}\left(s,a\right)=Q_{t}\left(s,a\right), & \left(s,a\right)\neq \left(s_{t},a_{t}\right), \end{array}\right. \end{array}$$

where $\alpha_{t}$ is a suitably chosen learning rate. Precisely speaking, the update Equation (1) corresponds to the asynchronous variant of Q-learning. The classical asymptotic performance analysis of Q-learning (in, for example, [36]) indicates that if (i) $\pi_{b}$ is exploratory enough such that all state–action pairs are visited infinitely often and (ii) ${\left(\alpha_{t}\right)}_{t\geq 0}$ satisfies the following conditions, known as the Robbins–Monro conditions [36,44]:

$$\alpha_{t}\geq 0,\;\sum_{t=0}^{\infty }\alpha_{t}=\infty ,\;\sum_{t=0}^{\infty }\alpha_{t}^{2}<\infty ,$$

then $Q_{t}\to_{t\to \infty }Q^{⋆}$ almost surely, for any choice of $Q_{0}\in R^{S\times A}$. For example, one such choice of the learning rate is as follows: for all t≥0, $\alpha_{t}=\frac{1}{N_{t}\left(s_{t},a_{t}\right)+1}$, where for any (s,a), $N_{t}\left(s,a\right)$ denotes the number of visits to (s,a) up to time *t*: $N_{t}\left(s,a\right)=\sum_{\tau =0}^{t-1}I\left\{\left(s_{\tau },a_{\tau }\right)=\left(s,a\right)\right\}$. The pseudo-code of Q-learning is described in Algorithm 1, where the learning rate sequence ${\left(\alpha_{t}\right)}_{t\geq 0}$ is considered as input.
**Algorithm 1** Q-learning [2].**Input:** dataset D, maximum iterations *T*, learning rates ${\left(\alpha_{t}\right)}_{t\geq 0}$**Initialization:** $Q_{0}=0\in R^{S\times A}$**for** t=0,1,…,T **do**   Sample action $a_{t}∼\pi_{b}\left(s_{t}\right)$ and observe $r_{t}∼R\left(s_{t},a_{t}\right)$ and $s_{t+1}∼P\left(\cdot |s_{t},a_{t}\right)$.   Compute $Q_{t+1}$ using (1).**end for**

It is worth remarking that some studies consider off-policy learning in the online setting, where data are collected from the environment while executing the algorithm. In such online settings, it is possible to choose $a_{t}$ according to an adaptive (randomized) policy $\pi_{t}$ (usually defined as a function of the current estimate $Q_{t}$), instead of sampling it from a fixed behavior policy. In doing so, the aim is to balance exploration and exploitation so as to collect higher rewards while learning the Q-function. A notable example is the ϵ-greedy policy, where at time *t*, $a_{t}$ is chosen greedily with respect to $Q_{t}\left(s_{t},\cdot \right)$ with probability 1−ϵ, and chosen uniformly at random (from A) with probability ϵ. In the theoretical part of this paper, we consider a fixed behavior policy.

### 3.3. Similarity and Equivalence Classes

We now present a definition of the equivalence structure considered in this paper. We start by stating the following definition of similarity in finite MDPs as introduced in [18]. A similar definition is provided in [23].

**Definition** **1** (Similar state–action pairs [18]). *Two state–action pairs (s,a) and $\left(s^{\prime },a^{\prime }\right)$ are called* **θ-similar** *if there exist mappings $\sigma_{s,a}:\left\{1,\ldots ,S\right\}\to S$ and $\sigma_{s^{\prime },a^{\prime }}:\left\{1,\ldots ,S\right\}\to S$ such that*

$$\begin{array}{c} \;∥P\left(\sigma_{s,a}\left(\cdot \right)|s,a\right)-P\left(\sigma_{s^{\prime },a^{\prime }}\left(\cdot \right)|s^{\prime },a^{\prime }\right){∥}_{1}\leq \theta . \end{array}$$

*We refer to $\sigma_{s,a}$ as the* **profile mapping** *(or for short,* **profile** *) for (s,a), and denote by $\sigma ={\left(\sigma_{s,a}\right)}_{s,a}$ the set of profile mappings across S×A.*

We stress that $\sigma_{s,a}$ in Definition 1 may not be unique in general. The case of 0-similarity is of particular interest: it is evident from Definition 1 that if (s,a) and $\left(s^{\prime },a^{\prime }\right)$ are 0-similar, then P(·|s,a) and $P(\cdot |s^{\prime },a^{\prime })$ are *identical up to some permutation* from S×A to S×A. Furthermore, 0-similarity induces a partition of the state–action space S×A as formalized below.

**Definition** **2** (Equivalence structure [18]). *0-similarity is an equivalence relation and induces a canonical partition of S×A. We refer to such a canonical partition as* **equivalence structure** *and denote it by C. We further define C:=|C|.*

**Figure 1:**
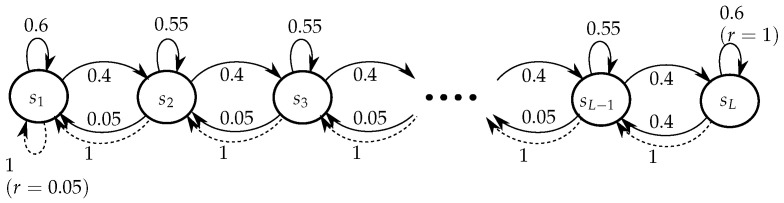
The *L*-state RiverSwim environment [45].

We provide an example to help understand Definition 2. Consider the RiverSwim environment [45] with 6 states and A={L,R} (see Figure 1). The two pairs $\left(s_{1},R\right)$ and $\left(s_{6},R\right)$ are 0-similar since $P\left(\cdot |s_{1},R\right)=\left[0.6,0.4,0,0,0,0\right]$ and $P\left(\cdot |s_{6},R\right)=\left[0,0,0,0,0.6,0.4\right]$, so there exist permutations $\sigma_{s_{1},R}$ and $\sigma_{s_{6},R}$ such that $P\left(\sigma_{s_{1},R}\left(\cdot \right)|s_{1},R\right)=P\left(\sigma_{s_{6},R}\left(\cdot \right)|s_{6},R\right)$. Additionally, all pairs $(s_{i},L),i=1,\ldots ,6$ are 0-similar, and so are $(s_{i},R),i=2,\ldots ,5$. We thus identify an equivalence structure C of S×A as follows: $C=\{c_{1},c_{2},c_{3}\}$ with $c_{1}=\left\{\left(s_{1},R\right),\left(s_{6},R\right)\right\}$, $c_{2}=\left\{\left(s_{i},R\right),i=2,\ldots ,5\right\}$, and $c_{3}=\left\{\left(s_{i},L\right),i=1,\ldots ,6\right\}$.

Note that for any finite MDP, Definition 2 trivially holds with C=S×A. There are many interesting environments that non-trivially admit the notion of equivalence structure in Definition 2. In such MDPs, it is often the case that the size *C* of the structure is much smaller than the size of the state–action space, i.e., C≪SA. For example, in a generic RiverSwim with *S* states, one has C=3. Another example admitting an equivalence structure is the classical grid-world MDP, which is detailed in Section 6.2.

**Off-policy learning in MDPs with equivalence structures.** In this work, we assume that the underlying MDP *M* admits an equivalence structure C as introduced above. In other words, the transition function *P* is such that S×A can be partitioned into C:=|C| classes, where the pairs in each class c∈C are 0-similar. We make the following assumption regarding the agent’s prior knowledge about C.

**Assumption** **A1.** *The agent has prior knowledge on C.*

Let c(s,a) denote the class that the pair (s,a) belongs to. Assumption A1 implies that the agent knows c(s,a) for any pair (s,a) and the associated profile mapping $\sigma_{s,a}$. Note, however, that the agent does not know the actual transition probabilities. Armed with such prior knowledge, we are interested in devising a model-free algorithm that is capable of leveraging the structure in *M* to improve the learning performance. We expect that the corresponding speed-up in learning the optimal Q-function could be significant in MDPs with C≪SA.

We also make the following assumption regarding the reward function to ease the presentation. (This assumption has often been made in the literature on theoretical RL (e.g., [6,46]) since the main challenge in RL arises from unknown transition probabilities.)

**Assumption** **A2**. *The agent knows the reward function R.*

## 4. The QL-ES Algorithm

In this section, we present a variant of Q-learning that exploits the equivalence structure in the environment to speed up the learning of the optimal Q-function. We call this algorithm QL-ES, which is short for ‘Q-learning with equivalence structure’.

QL-ES follows the same machinery of QL but is also built on the idea that the knowledge on C and the corresponding profile mappings allows for using the triplet $\left(s_{t},a_{t},s_{t+1}\right)$ collected at each time *t* to update potentially multiple entries of $Q_{t}$. Precisely speaking, the Q-function update for a given pair (s,a) requires a sample from R(s,a) and a sample from P(·|s,a). Since the agent perfectly knows C, it can determine $c(s_{t},a_{t})$, i.e., the class $\left(s_{t},a_{t}\right)$ belongs to. Hence, it knows all other pairs belonging to the same class as $\left(s_{t},a_{t}\right)$. Then using $s_{t+1}$ (sampled from $P(\cdot |s_{t},a_{t})$) at time *t*, the agent can construct samples for all other pairs in $c(s_{t},a_{t})$ as follows. If $\left(s,a\right)\in c\left(s_{t},a_{t}\right)$, then there is a mapping $\sigma_{s,a}$ and a state $s_{t+1}^{\left(sa\right)}$ such that $P\left(\sigma_{s_{t},a_{t}}\left(s_{t+1}\right)|s_{t},a_{t}\right)=P\left(\sigma_{s,a}\left(s_{t+1}^{\left(sa\right)}\right)|s,a\right)$. In other words, the sample $s_{t+1}$ obtained from $P(\cdot |s_{t},a_{t})$ is equivalent to obtaining a fresh sample $s_{t+1}^{\left(sa\right)}$ from P(·|s,a). As $\sigma_{s,a}$ and $\sigma_{s_{t},a_{t}}$ are known, the agent finds $s_{t+1}^{\left(sa\right)}:=\sigma_{s,a}^{-1}\left(\sigma_{s_{t},a_{t}}\left(s_{t+1}\right)\right)$, where $\sigma^{-1}$ denotes the inverse mapping of σ. In other words, $s_{t+1}^{\left(sa\right)}$ acts as a *counterfactual next-state* for $\left(s,a\right)\in c\left(s_{t},a_{t}\right)$ thanks to the knowledge on C.

The agent thus uses $\left(s_{t},a_{t},s_{t+1}\right)$ to update $Q_{t}$ for all $\left(s,a\right)\in c\left(s_{t},a_{t}\right)$. In summary, we update $Q_{t}$ as follows: For all t≥0,

(2) $$\begin{array}{c} Q_{t+1}\left(s_{t},a_{t}\right)=\left\{\begin{array}{cc} \left(1-\alpha_{t}\right)Q_{t}\left(s,a\right)+\alpha_{t}\left(\bar{R}\left(s,a\right)+\gamma \max_{a^{\prime }\in A}Q_{t}\left(s_{t+1}^{\left(sa\right)},a^{\prime }\right)\right), & \left(s,a\right)\in c\left(s_{t},a_{t}\right),\\ Q_{t+1}\left(s,a\right)=Q_{t}\left(s,a\right), & \left(s,a\right)\notin c\left(s_{t},a_{t}\right), \end{array}\right. \end{array}$$

where ${\left(\alpha_{t}\right)}_{t\geq 0}$ is a sequence of suitably chosen learning rates, as in (1). The pseudo-code of QL-ES is provided in Algorithm 2.
**Algorithm 2** QL-ES**Input:** dataset D, maximum iterations *T*, learning rates ${\left(\alpha_{t}\right)}_{t\geq 0}$, equivalence structure C**Initialization:** $Q_{0}=0\in R^{S\times A}$.**for** t=0,1,2,…,T **do**    Sample action $a_{t}∼\pi_{b}\left(s_{t}\right)$ and observe $s_{t+1}∼P\left(\cdot |s_{t},a_{t}\right)$.    Find $c(s_{t},a_{t})$.    **for** $\left(s,a\right)\in c\left(s_{t},a_{t}\right)$ **do**        $s_{t+1}^{\left(sa\right)}=\sigma_{s,a}^{-1}\left(\sigma_{s_{t},a_{t}}\left(s_{t+1}\right)\right)$     Compute $Q_{t+1}$ using (2)   **end for****end for**

When the underlying MDP admits some equivalence structure, QL-ES performs multiple updates of Q-function at any slot, in contrast to structure-oblivious Q-learning that updates only the Q-function of the current state–action pair. Thus, we expect learning the optimal Q-function under QL-ES to be faster than Q-learning; this will be corroborated by the numerical experiments in Section 6. It is also worth mentioning that QL-ES is never worse than Q-learning, as for the trivial partition C=S×A, which holds for any finite MDP, QL-ES reduces to Q-learning.

**Remark** **1.** *The multiple updates used in QL-ES can be straightforwardly combined with many other variants of Q-learning, such as Speedy Q-learning [46] and UCB-QL [41].*

We finally remark that some works in the literature on Q-learning use learning rates of the form $\alpha_{t}=f\left(N_{t}\left(s_{t},a_{t}\right)\right)$, where $N_{t}\left(s,a\right)=\sum_{\tau =0}^{t-1}I\left\{\left(s_{\tau },a_{\tau }\right)=\left(s,a\right)\right\}$ and where *f* is some suitable function *f* satisfying the Robbins–Monro conditions, e.g., $\alpha_{t}=\frac{1}{N_{t}\left(s_{t},a_{t}\right)+1}$. Such learning rates in the case of QL-ES can be modified to $\alpha_{t}=f\left(N_{t}\left(c\left(s_{t},a_{t}\right)\right)\right)$, where for any c∈C, $N_{t}\left(c\right):=\sum_{\tau =0}^{t-1}I\left\{\left(s_{\tau },a_{\tau }\right)\in c\right\}$.

## 5. Theoretical Guarantee for QL-ES

In this section, we investigate the theoretical guarantee of QL-ES in terms of sample complexity in the PAC setting. Specifically, we are interested in characterizing the deviation between the optimal Q-function $Q^{⋆}$ and its estimate $Q_{T}$ computed by QL-ES after *T* time steps. A relevant notion of deviation often studied in the literature (see, e.g., [37,38]) is the $\ell_{\infty }$-distance between $Q_{T}$ and $Q^{⋆}$:

(3) $$∥Q^{⋆}-Q_{T}{∥}_{\infty }=\max_{s,a}\left|Q^{⋆}\left(s,a\right)-Q_{T}\left(s,a\right)\right|$$

which captures the worst error (with respect to $Q^{⋆}$) among various pairs. One may study the rate at which the error function $∥Q^{⋆}-Q_{T}{∥}_{\infty }$ decays as a function of *T*. Alternatively, one may characterize *the PAC sample complexity* defined as the number *T* of steps needed until $Q_{T}$ satisfies $∥Q^{⋆}-Q_{T}{∥}_{\infty }\leq \varepsilon$ with probability at least 1−δ, for pre-specified ε and δ. We consider the latter case.

Let us first recall the classic definition of cover time $t_{\mathrm{cover}}$, which is a standard notion in the literature on Markov chains as well as those studying theoretical guarantees of Q-learning (and its variants) [9,38,39]. Let $t_{1}\geq 0$ and let $t_{2}>t_{1}$ denote the *first* time step such that all state–action pairs are visited at least once with probability at least $\frac{1}{2}$. Then, the cover time $t_{\mathrm{cover}}$ is defined as the maximum value of $t_{2}-t_{1}$ over all initial pairs $\left(s_{t_{1}},a_{t_{1}}\right)$. Note that $t_{\mathrm{cover}}$ depends on both the MDP *M* and the behavior policy $\pi_{b}$. More precisely, it depends on the mixing properties of the Markov chain induced by $\pi_{b}$ on *M*. Further, we have $t_{\mathrm{cover}}\geq SA$.

Next, we introduce a notion of cover time for equivalence classes, which is relevant to the performance analysis of QL-ES. We believe it can be of independent interest.

**Definition** **3.** *Let M be a finite MDP and C be an equivalence structure in M. Given $t_{1}\geq 0$, let $t_{2}>t_{1}$ denote the first time step such that for each c∈C, some state–action pair in c is visited at least once with probability at least $\frac{1}{2}$. Then, the cover time with respect to the equivalence structure C in M, denoted by $t_{\mathrm{cover},C}$, is defined as the maximum value of $t_{2}-t_{1}$ over all initial choices of $c(s_{t_{1}},a_{t_{1}})$ (i.e., the class the initial pair $\left(s_{t_{1}},a_{t_{1}}\right)$ belongs to).*

The following theorem provides a non-asymptotic sample complexity for QL-ES. It concerns constant learning rates, i.e., $\alpha_{t}=\alpha$ for all t≥0, where α may depend on ε and δ, among other things.

**Theorem** **1.** *There exist some universal constants $\kappa_{0},\kappa_{1}$ such that for any δ∈(0,1) and $\varepsilon \in (0,\frac{1}{1-\gamma }]$, we have $∥Q^{⋆}-Q_{T}{∥}_{\infty }\leq \varepsilon$ with probability greater than 1−δ, provided that the number T of steps and learning rate α jointly satisfy*

$$\begin{array}{c} T\geq \frac{\kappa_{0}t_{\mathrm{cover},C}}{{\left(1-\gamma \right)}^{5}\varepsilon^{2}}\log^{2}\left(\frac{CT}{\delta }\right)\log\left(\frac{1}{{\left(1-\gamma \right)}^{2}\varepsilon }\right),\;\alpha =\min\left\{\frac{\kappa_{1}{\left(1-\gamma \right)}^{4}\varepsilon^{2}}{\gamma^{2}\log\left(CT/\delta \right)},\;\frac{1}{2}\right\}\;. \end{array}$$

A proof of this theorem is provided in Appendix A. Our proof is an adaptation of the of the proof of Theorem 2 in [9], which concerns the sample complexity of Q-learning.

**Comparison with sample complexity of Q-learning.** Theorem 1 tells us that the number of steps to have $∥Q^{⋆}-Q_{T}{∥}_{\infty }\leq \varepsilon$ with high probability depends on $t_{\mathrm{cover},C}\varepsilon^{-2}{\left(1-\gamma \right)}^{-5}$ (up to some logarithmic factors), where $t_{\mathrm{cover},C}$, defined in Definition 3, is the cover time with respect to C. Comparing this result against the sample of complexity of Q-learning (e.g., Theorem 2 in [9]) reveals that using QL-ES yields an improvement over Q-learning by a factor of const.×ξ, where

$$\xi :=\xi \left(M,C,\pi_{b}\right):=\frac{t_{\mathrm{cover}}}{t_{\mathrm{cover},C}}\;.$$

This ratio ξ is a problem-dependent constant (depending on both *M* and (C,σ)). It also depends on the behavior policy $\pi_{b}$ in view of the definitions of the cover times. It is evident that ξ≥1 for any choice of *M* and C. For a given MDP *M*, the ratio $\xi (M,C,\pi_{b})$ can be numerically computed; we report numerical values of ξ for several domains in Section 6.3. On the other hand, deriving the analytical bounds on the ratio $\xi (M,C,\pi_{b})$ for any *M* appears to be complicated and tedious, if possible at all. Nonetheless, it is possible to construct simple problem instances, where one can derive analytical bounds on ξ.

**Figure 2:**
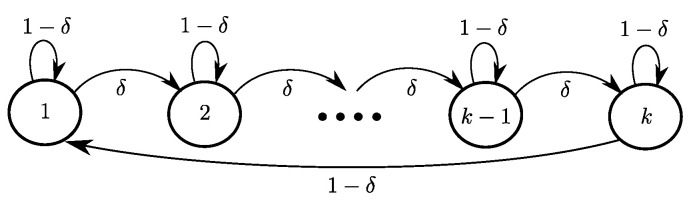
An illustrative example where ξ=O(S).

Figure 2 portrays one such example; this example is a simple Markov chain but can be easily extended to become an MDP. Easy calculations show that $t_{\mathrm{cover}}=\left(S-1\right)/\delta$ and $t_{\mathrm{cover},C}=1$ so that ξ=(S−1)/δ. Hence, one here has ξ=O(S). This simple example demonstrates that the gain of QL-ES over Q-learning in some domains could be as large as O(S), the size of the state space. Additionally, Theorem 1 reveals that in such domains, the theoretical sample complexity bound of QL-ES does not depend on *S* but on *C*, the number of classes in C.

We refer the reader to the results in Section 6.3, where we present numerical bounds on ξ in some MDPs, which serve as the standard domain in the RL literature.

## 6. Simulation Results

This section is devoted to reporting numerical experiments conducted to examine the performance of QL-ES against the (structure-oblivious) Q-learning algorithm. First, we present the considered evaluation metrics and environments. Then we present numerical assessment of ξ for these environments. Finally, we report empirical sample complexities of QL-ES and Q-learning in the environments.

### 6.1. Evaluation Metrics

We consider two evaluation metrics in the experiments:

(i) *Max-norm Q-value Error* defined as $∥Q^{⋆}-Q_{t}{∥}_{\infty }$;
(ii) *Total Policy Error* defined as $∥\pi^{⋆}-\pi_{t}^{\mathrm{greedy}}{∥}_{1}$, where $\pi_{t}^{\mathrm{greedy}}$ denotes the greedy policy w.r.t. $Q_{t}$, i.e., $\pi_{t}^{\mathrm{greedy}}\left(s\right):=\arg\max_{a}Q_{t}\left(s,a\right)$ for all *s*.

The metric (i), which is in line with the definition of sample complexity studied in Section 5, captures the maximum difference between $Q_{t}$ and $Q^{⋆}$ over all state–action pairs and allows us to empirically study the convergence speed of $Q_{t}$ toward $Q^{⋆}$. The second metric captures the quality of the estimate $Q_{t}$ in terms of inferred policies. Evidently, the quantity $∥\pi^{⋆}-\pi_{t}^{\mathrm{greedy}}{∥}_{1}$ returns the number of states at which $\pi_{t}^{\mathrm{greedy}}$ prescribes a sub-optimal action. Hence, the metric (ii) may capture how bad the policy derived from $Q_{t}$ (i.e., $\pi_{t}^{\mathrm{greedy}}$) would be, compared to $\pi^{⋆}$, had we stopped at time step *t*. Equivalently, we may compute the metric (ii) via

(4) $$\sum_{s\in S}I\left\{Q_{t}\left(s,\pi^{⋆}\left(s\right)\right)-\max_{a\neq \pi^{⋆}\left(s\right)}Q_{t}\left(s,a\right)<0\right\}\;.$$

Working with (4) is preferred, as then, one may not worry about how ties (in argmax) are broken when either $\pi_{t}$ or $\pi^{⋆}$ is not unique.

### 6.2. Environments

We consider two environments: *RiverSwim* and *GridWorld*. These are classical MDPs widely used in the RL literature. Both render suitability to demonstrate the numerical performance of QL-ES since each allows us to define a family of MDPs with progressive difficulty levels.

**RiverSwim and variants.** A generic RiverSwim MDP with *L* states is shown in Figure 1, which extends the classical 6-state RiverSwim presented in [45]. This MDP is constructed so that efficient exploration is required to obtain the optimal policy. The larger the number *L* of states, the more exploration is required. The *L*-state RiverSwim (with L≥3) admits an equivalence structure with C=3 regardless of *L*. We consider RiverSwim instances with various *L* so as to have MDPs with progressive difficulty levels while having a fixed number of classes. In some experiments, we consider a slightly modified version of RiverSwim, which we shall call *Perturbed RiverSwim*. It is identical to RiverSwim (Figure 1) except that in any state $s_{i}$, where i<L is even, $p(s_{i}|s_{i},R)=0.65$ and $p(s_{i+1}|s_{i},R)=0.3$. It is clear that there are C=4 classes in a *L*-state Perturbed RiverSwim.

**Figure 3:**
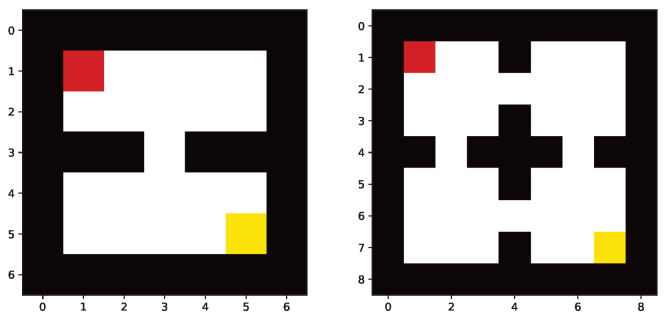
The 2-room grid world (**left**) and 4-room grid world (**right**) with walls in black, initial state in red, and goal state in yellow.

**GridWorld.** We also consider 2-room and 4-room grid-world MDPs with different grid sizes. Figure 3 shows a 7×7 2-room and a 9×9 4-room grid-worlds, respectively. In both environments, the agent starts at the upper-left corner (in red) and is supposed to reach the lower-right corner (in yellow), where it is given a reward of 1 and then sent back to the initial red state. At each step, the agent has four possible actions (hence, A=4): Going up, left, down, or right. Black squares indicate the wall where the agent is not able to penetrate through. After executing a given action, the agent has a probability of 0.1 to stay in the same state, has a probability of 0.7 to move to the desired direction, and has a probability of 0.06 and 0.14 to move to the other two possible directions. If the wall blocks the agent, it stays where it is, and the transition probability of the next state is added to that of the current state.

It is clear that the grid-world MDPs above admit some equivalence structure. In the case of 2-room (respectively, 4-room), the state–action space is of size 84 (respectively, 160), while the number of classes remains 8 in both. In Table 1, we also present six examples of grid-world environments with walls defined according to the way mentioned above. In the introduced 2-room and 4-room MDPs, the number of state–action pairs changes with the increase in the grid size, while the number of classes remains fixed.

### 6.3. Bounds on the Ratio ξ

We recall from Section 5 that the theoretical gain of QL-ES over Q-learning in terms of sample efficiency is captured by the problem-dependent quantity $\xi =\frac{t_{\mathrm{cover}}}{t_{\mathrm{cover},C}}$. In this subsection, we compute ξ for the introduced environments with the aim of providing insights into the growth of ξ as the number *S* of states grows. Specifically, we consider RiverSwim, Perturbed RiverSwim (introduced in Section 6.2), and GridWorld MDPs, each with growing number of states. In each case, we report empirical values for $t_{\mathrm{cover}}$ and $t_{\mathrm{cover},C}$ together with the corresponding 95% confidence intervals. The empirical $t_{\mathrm{cover}}$ is computed as the median value (across 100 independent runs for every possible initial state-action pair) of the number of steps it takes to discover all state-action pairs starting from a given initial state-action pair. A similar procedure is used for $t_{\mathrm{cover},C}$.

Table 2, Table 3 and Table 4 summarize empirical values of $t_{\mathrm{cover}}$ and $t_{\mathrm{cover},C}$ (together with the associated 95% confidence intervals denoted by CI) for RiverSwim, Perturbed RiverSwim, and 2-room GridWorld, respectively, with varying number of states in each case. In the case of GridWorld, we ran a uniform agent (sampling each action uniformly), wheres in RiverSwim MDPs, the agent samples R (resp. L) with probability 0.8 (resp. 0.2).

These results reveal that $t_{\mathrm{cover},C}$ is much smaller than $t_{\mathrm{cover}}$ in all cases. Furthermore, they indicate that while $t_{\mathrm{cover}}$ grows rapidly as *S* increases (in any family of the MDPs considered), $t_{\mathrm{cover},C}$ experiences a much smaller growth. As for the ratio ξ, we report $\xi_{\mathrm{LCB}}$ as the lower confidence bound obtained by dividing the lower value in the CI for $t_{\mathrm{cover}}$ by the upper value in the CI for $t_{\mathrm{cover},C}$. This is a rather conservative estimate of the true ξ but ensures that $\xi \leq \xi_{\mathrm{LCB}}$ with probability at least 0.9. The reported values demonstrate that in these environments, ξ grows rapidly as the size of state space grows. This observation verifies that the theoretical gain of QL-ES over Q-learning can be significant.

### 6.4. Experimental Results with Exact Equivalence Structure

We now turn to reporting experimental results for QL-ES and Q-learning in RiverSwim and GridWorld. In the following figures, QL indicates the standard Q-learning algorithm (Algorithm 1). We used a constant learning rate α=0.05 and ϵ-greedy policy (with values of ϵ to be specified later). Furthermore, all the results are averaged over 100 independent runs, and the corresponding 95% confidence intervals are shown.

**Figure 4:**
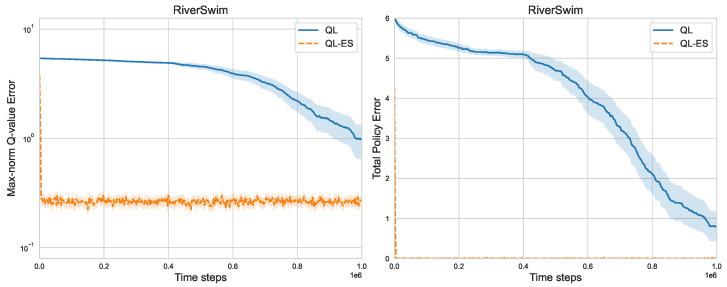
Results in 6-state RiverSwim.

Figure 4 presents the max-norm Q-value error and total policy error under both QL-ES and Q-learning in a 6-state RiverSwim, where we set γ=0.9 and ϵ=0.5. It is evident that QL-ES significantly outperforms Q-learning. The Q-value error under Q-learning decays at a very slow rate until about about $4\times 10^{5}$ steps. After this step, the decay rate increases tangibly. In contrast, the Q-value error under QL-ES decays at a much faster speed. Under Q-learning, the total policy error remains above 5 until time step $4\times 10^{5}$, which implies that only one state has learned its optimal policy. On the contrary, the total policy error under QL-ES drops to the vicinity of 0 very quickly. These results verify that the empirical gain of leveraging the equivalence structures in MDPs, in terms of the number of samples, can be significant.

**Figure 5:**
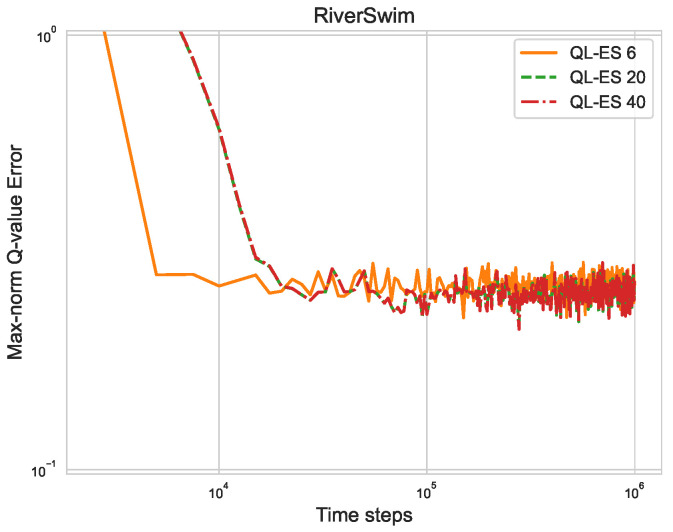
Comparison between RiverSwim domains.

To demonstrate the scalability of QL-ES, in Figure 5, we present the Q-value error under QL-ES in RiverSwim instances with 6, 20, and 40 states. As the figure shows, although the error in the 6-state RiverSwim starts decaying much earlier than the others, all of them exhibit a similar rate of decay. Moreover, the curves corresponding to 20-state and 40-state instances are almost indistinguishable. This result showcases MDPs where the sample complexity of QL-ES does not scale with the size of the state–action space and is mostly determined by the number of classes.

**Figure 6:**
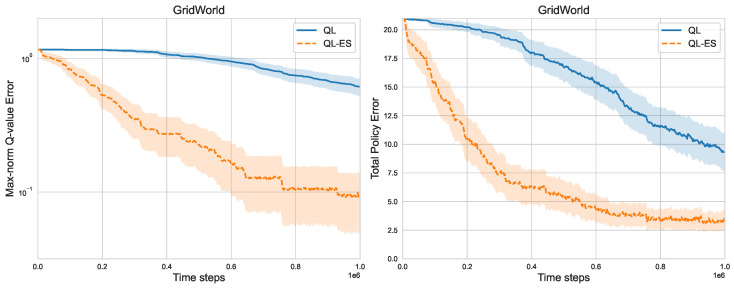
Results in 2-room grid world.

**Figure 7:**
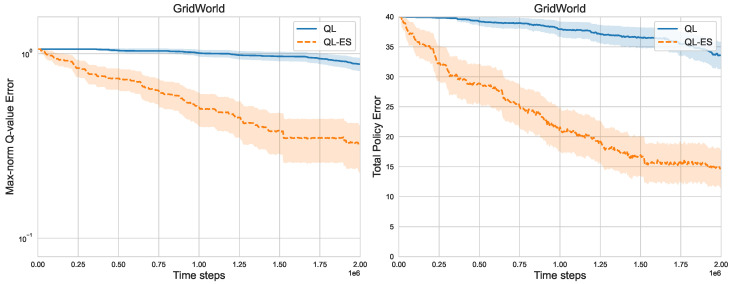
Results in 4-room grid world.

We now turn to the results for GridWorld MDPs. Figure 6 and Figure 7 show the results for QL-ES and Q-learning in 2-room and 4-room GridWorld MDPs, where we used γ=0.85 and ϵ=0.2 in the 2-room and γ=0.85 and ϵ=0.3 in the 4-room.

As in RiverSwim MDPs, QL-ES significantly outperforms Q-learning in the grid-world environments. For Q-learning, the Q-value error remains considerable, even for $10^{6}$ samples. Although both QL-ES and Q-learning do not fully learn an optimal policy by the end of the run, the total policy error decays much faster under QL-ES. Overall, the results demonstrate that exploiting equivalence structure is beneficial in grid-world MDPs. Comparing Figure 6 and Figure 7, it is evident that QL-ES still obtains relatively better performance than Q-learning with the increase in state space. Moreover, similar trends are expected when conducting this experiment in larger grid-world MDPs.

### 6.5. The Gain in the Case of θ-Similar Pairs

**Figure 8:**
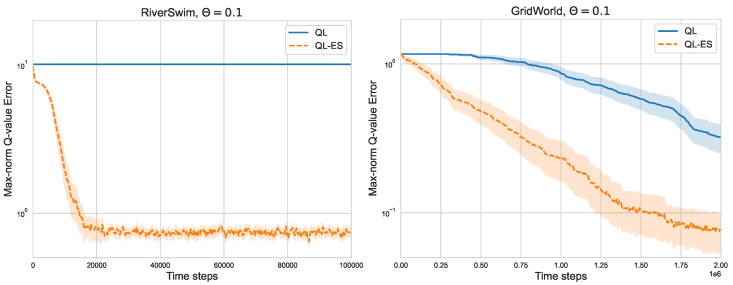
Results with θ-similar pairs: Modified RiverSwim (**left**) and Modified GridWorld (**right**).

We now investigate the case where the MDP may not admit any equivalence structure but admits θ-similarity across its state–action space; see Definition 1. To this effect, we introduce *Modified RiverSwim* and *Modified GridWorld* defined as follows. The *Modified RiverSwim* is identical to 6-state RiverSwim (Figure 1), except that its non-zero transition probabilities under $\left(s_{2},R\right)$ and $\left(s_{4},R\right)$ are changed from [0.05,0.55,0.4] to [0.15,0.3,0.55]. The *Modified GridWorld* is identical to 7×7 2-room grid-world, except that the non-zero transition probabilities under ($s_{1}$,down), ($s_{5}$,down), and ($s_{7}$,down) are set to [0.6,0.05,0.2,0.15] instead of [0.7,0.06,0.14,0.1]. Modified RiverSwim admits an equivalence structure, but we remark that it satisfies θ-similarity with θ=0.1. Similarly, Modified GridWorld satisfies θ-similarity with θ=0.1. Figure 8 shows the results in Modified RiverSwim (with γ=0.95) and Modified GridWorld (γ=0.85). It is evident that in both cases, QL-ES still achieves smaller Q-value error than Q-learning.

### 6.6. The Impact of Partially Using the Structure

Considering MDPs with huge state–action spaces, it is necessary to take into account the feasibility of using only a few equivalent pairs. This thus naturally leads to the question as to whether only using a few equivalent pairs would lead to a reasonable performance gain. Therefore, here we investigate the convergence speed when choosing *a subset* of equivalent state–action pairs at each time step, rather than considering all the state–action pairs in the same class.

**Figure 9:**
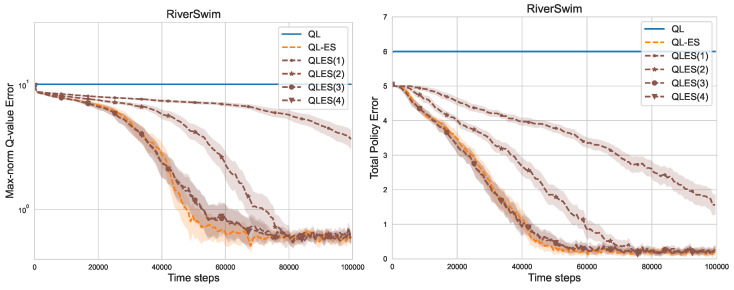
Results in RiverSwim: S=6.

As shown in Figure 9, we can still obtain reasonable performance only considering a few equivalent pairs. The numbers in brackets represent how many equivalent pairs are used. In RiverSwim with 6 states (SA=12), the performance of QLES(3) and QLES(4) is comparable to QL-ES. Interestingly, we already observe a significant improvement over Q-learning using QLES(1), i.e., when using only one additional observation in the Q-learning update.

Meanwhile, the total policy error is less than or close to one. This shows that the optimal policy is correctly learned in almost all the states.

**Figure 10:**
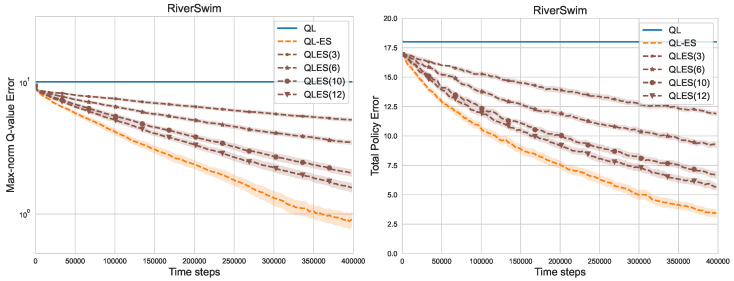
Results in RiverSwim: S=20.

In 20-state RiverSwim (SA=40), algorithms with few equivalent pairs can still achieve excellent performance, albeit not as good as QL-ES. Additionally, as Figure 10 shows, using more equivalent pairs leads to better sample efficiencies in MDPs with large state space. Concerning QLES(3)-QLES(12), the Q-value error gets smaller when more equivalent pairs are used.

From the perspective of policy, even QLES(3) and QLES(6) manage to find optimal actions significantly faster than Q-learning. In addition, QLES(10) is far superior to QLES(6) because of the quite smaller Q-value error and total policy error. The results show that algorithms with a suitable number of equivalent pairs are sufficient to learn an optimal policy in most states reasonably fast.

## 7. Conclusions

We studied off-policy learning in discounted Markov decision processes, where some equivalence structure exists in the state–action space. We presented a model-free algorithm called QL-ES, which is a natural extension of the classical asynchronous Q-learning but capable of exploiting the equivalence structure. We presented a high-probability sample complexity bound for QL-ES, and discussed how it improves that of Q-learning. As demonstrated, there exist problem instances on which the improvement over Q-learning could be a multiplicative factor of *S*, the size of the state space. Through extensive numerical experiments in standard domains, we demonstrated that QL-ES significantly improves over (structure-oblivious) Q-learning. These results revealed that exploiting state–action equivalence favors faster convergence of the Q-function and policy learning in large MDPs. A limitation of our approach is the need for the prior knowledge on the structure. To the best of our knowledge, existing methods for learning the equivalence structure are all model-based. Hence, an interesting question is whether it is possible to exploit the equivalence structure *without prior knowledge on the structure using only model-free algorithms*. Devising such model-free algorithms (or otherwise establishing an impossibility result) is an interesting yet challenging topic for future work. Another interesting direction for future work is to investigate ways to combine the knowledge of the equivalence structure with function approximation methods. Finally, another avenue for future work is to study model-free algorithms for the regret minimization setting in average-reward MDPs (e.g., [47,48]).

## Figures and Tables

**Table 1:** State–action space and equivalence classes comparison in grid-world MDPs.

Environment	States	7×7	9×9	11×11	20×20	50×50	100×100
2-room	SA	84	172	292	1228	9028	$3.8\times 10^{4}$
2-room	*C*	8	8	8	8	8	8
4-room	SA	80	160	272	1172	8852	$3.7\times 10^{4}$
4-room	*C*	8	8	8	8	8	8

**Table 2:** Empirical values of $t_{\mathrm{cover}}$, $t_{\mathrm{cover},C}$, and $\xi_{\mathrm{LCB}}$ for RiverSwim with *S* states.

*S*	6	10	14	20
$t_{\mathrm{cover}}$	131, CI=[97,158]	513, CI=[340,658]	2529, CI=[1867,3196]	12,792, CI=[7577,15,688]
$t_{\mathrm{cover},C}$	12, CI=[9,16]	33, CI=[26,38]	56, CI=[46,66]	113, CI=[94,133]
$\xi_{\mathrm{LCB}}$	$\frac{97}{16}\approx 6.1$	$\frac{340}{38}\approx 8.9$	$\frac{1867}{66}\approx 28.3$	$\frac{7577}{133}\approx 57.0$

**Table 3:** Empirical values of $t_{\mathrm{cover}}$, $t_{\mathrm{cover},C}$, and $\xi_{\mathrm{LCB}}$ for Perturbed RiverSwim with *S* states.

*S*	6	10	14	20
$t_{\mathrm{cover}}$	116, CI=[97,136]	557, CI=[351,655]	2011, CI=[1616,2451]	11,856, CI=[7282,17,103]
$t_{\mathrm{cover},C}$	14, CI=[12,17]	32, CI=[23,37]	58, CI=[46,67]	114, CI=[99,130]
$\xi_{\mathrm{LCB}}$	$\frac{97}{17}\approx 5.7$	$\frac{351}{37}\approx 9.5$	$\frac{1616}{67}\approx 24.1$	$\frac{7282}{130}\approx 56.0$

**Table 4:** Empirical values of $t_{\mathrm{cover}}$, $t_{\mathrm{cover},C}$, and $\xi_{\mathrm{LCB}}$ for 2-room GridWorld with *S* states.

*S*	21	43	71	111
$t_{\mathrm{cover}}$	2164, CI=[1939,2355]	5480, CI=[4890,5889]	11,310, CI=[9817,12,735]	22,793, CI=[20,571,24,882]
$t_{\mathrm{cover},C}$	205, CI=[125,251]	418, CI=[329,481]	877, CI=[613,1011]	1338, CI=[1101,1551]
$\xi_{\mathrm{LCB}}$	$\frac{1939}{251}\approx 7.7$	$\frac{4890}{481}\approx 10.2$	$\frac{9817}{1011}\approx 9.7$	$\frac{20571}{1551}\approx 13.3$

## Data Availability

Not applicable.

## Appendix A. Sample Complexity of QL-ES: Proof of Theorem 1

The following proof for Theorem 1 is a direct adaptation from Theorem 2 of [9]. We closely follow the notations and definitions used in [9].

### Appendix A.1. Notations

Before presenting the result and going into the details of the proof, some notations must be introduced. In the following, letters in bold, such as Q, shall stand for matrices and vectors, and for a matrix $M\in R^{N\times L}$, M(i) is the *i*-th row of M. Additionally, as long as there is no confusion, applying operations such as $\sqrt{\cdot }$ or |·| to a matrix is the entry-wise operation: for example, $\left|M\right|=\left(\right|m_{ij}{\left|\right)}_{i,j}$. Finally, 1 is the vector full of ones, and I is the identity matrix.

Moreover, the following quantities appear in the proof:

(A1) $$\begin{array}{cc} \; & t_{\mathrm{cover},\mathrm{all}}=t_{\mathrm{cover},C}\log\left(\frac{T}{\delta }\right) \end{array}$$

(A2) $$\begin{array}{cc} \; & t_{\mathrm{th}}=\max\left\{\frac{2t_{\mathrm{cover},\mathrm{all}}}{\alpha }\log\left(\frac{1}{{\left(1-\gamma \right)}^{2}\varepsilon }\right),t_{\mathrm{cover},\mathrm{all}}\right\} \end{array}$$

(A3) $$\begin{array}{cc} \; & \rho =\left(1-\gamma \right)(1-{\left(1-\alpha \right)}^{\frac{1}{2}}) \end{array}$$

Let us introduce the short-hand $c_{t}:=c\left(s_{t},a_{t}\right)$ for any t≥0. In order to analyze the update in QL-ES in a matrix-form way, we introduce

(A4) $$\begin{array}{cc} \; & \Lambda_{t}\left(\left(s,a\right),\left(s,a\right)\right)=\left\{\begin{array}{cc} \alpha & \mathrm{if}\;\left(s,a\right)\in c_{t-1}\\ 0 & \mathrm{otherwise} \end{array}\right. \end{array}$$

(A5) $$\begin{array}{cc} \; & P_{t}\left(\left(s,a\right),s^{\prime }\right)=\left\{\begin{array}{cc} 1 & \mathrm{if}\;\left(s,a\right)\in c_{t-1}\;\mathrm{and}\;s^{\prime }=\sigma_{s,a}^{-1}\left(\sigma_{s_{t-1},a_{t-1}}\left(s_{t}\right)\right)\\ 0 & \mathrm{otherwise} \end{array}\right. \end{array}$$

and we will use $Q_{t}$ for the Q-value matrix (of size S×A) and P for the transition matrix (of size SA×S). Notice that $\Lambda_{t}\in {\left[0,1\right)}^{SA\times SA}$ is diagonal, and $P_{t}\in {\left[0,1\right]}^{SA\times S}$. Then we have:

$$\begin{array}{c} Q_{t}=\left(I-\Lambda_{t}\right)Q_{t-1}+\Lambda_{t}\left(r+\gamma P_{t}V_{t-1}\right)\;, \end{array}$$

where $V_{t}\in R^{S}$ is the vector of value estimates at time *t*, whose *s*-th element is given by $\max_{a}Q_{t}\left(s,a\right)$. Finally, we are interested in bounding $\Delta_{t}=Q_{t}-Q^{⋆}.$

The following lemma showcases the role of the cover time, and the proof is given in Appendix B.1:

**Lemma** **A1.**
*Define the event*

$$K_{l}=\left\{\exists c\in C\;s.t.\;c\;is\;not\;visited\;within\;iterations\;\left(lt_{cover,all},\left(l+1\right)t_{cover,all}\right]\right\}$$

*and $L=\lfloor \frac{T}{t_{cover,all}}\rfloor$. Then: $P\left(\cup_{l=0}^{L}K_{l}\right)\leq \delta$.*

### Appendix A.2. Proof of Theorem 1

As established in the proof of [9] (Theorem 2) (see Equation (39) there), we have

$$\Delta_{t}=\left(I-\Lambda_{t}\right)\Delta_{t-1}+\gamma \Lambda_{t}\left(P_{t}-P\right)V^{⋆}+\gamma \Lambda_{t}P_{t}\left(V_{t-1}-V^{⋆}\right),$$

which, using an inductive argument, yields

$$\begin{array}{c} \Delta_{t}=\underset{\beta_{1,t}}{\underbrace{\gamma \sum_{i=1}^{t}\prod_{j=i+1}^{t}\left(I-\Lambda_{j}\right)\Lambda_{i}\left(P_{i}-P\right)V^{⋆}}}+\underset{\beta_{2,t}}{\underbrace{\gamma \sum_{i=1}^{t}\prod_{j=i+1}^{t}\left(I-\Lambda_{j}\right)\Lambda_{i}P_{i}\left(V_{i-1}-V^{⋆}\right)}}+\underset{\beta_{3,t}}{\underbrace{\prod_{j=1}^{t}\left(I-\Lambda_{j}\right)\Delta_{0}}}. \end{array}$$

Hence, $\left|\Delta_{t}\right|\leq \left|\beta_{1,t}\right|+\left|\beta_{2,t}\right|+\left|\beta_{3,t}\right|$, where ≤ holds element-wise. Furthermore, as in the proof of [9] (Theorem 2),

$$\begin{array}{c} {∥P_{i}\left(V_{i-1}-V^{⋆}\right)∥}_{\infty }\leq {∥P_{i}∥}_{1}{∥V_{i-1}-V^{⋆}∥}_{\infty }={∥Q_{i-1}-Q^{⋆}∥}_{\infty }={∥\Delta_{i-1}∥}_{\infty }, \end{array}$$

since ${∥P_{i}∥}_{1}=1$ in view of the definition of $P_{i}$. Hence,

(A6) $$\begin{array}{c} \left|\beta_{2,t}\right|\leq \gamma \sum_{i=1}^{t}{∥\Delta_{i-1}∥}_{\infty }\prod_{j=i+1}^{t}\left(I-\Lambda_{j}\right)\Lambda_{i}1\;. \end{array}$$

The following two lemmas provide upper bounds on $|\beta_{1,t}|$ and $|\beta_{3,t}|$.

**Lemma** **A2.**
*There exists a universal constant κ>0 such that, for any 0<δ<1,*

(A7)
$$\begin{array}{c} \forall \;1\leq t\leq T,\;\left|\beta_{1,t}\right|\leq \tau_{1}{∥V^{⋆}∥}_{\infty }1 \end{array}$$

*with probability at least 1−δ, where $\tau_{1}=\kappa \gamma \sqrt{\alpha \log\left(\frac{CT}{\delta }\right)}$.*

**Lemma** **A3.**
*For all t>0, we have $\left|\beta_{3,t}\right|\leq {∥\Delta_{0}∥}_{\infty }1$. Furthermore, with probability at least 1−δ,*

$$\begin{array}{cc} \forall \;t\in \left[t_{cover,all},T\right],\;\left|\beta_{3,t}\right|\; & \leq {\left(1-\alpha \right)}^{\frac{t}{2t_{cover,all}}}{∥\Delta_{0}∥}_{\infty }1. \end{array}$$

Lemma A2 is proven in Appendix B.2. The proof of Lemma A3 is the same as the proof of [9] (Lemma 6) but using appropriate definitions of $t_{\mathrm{cover},\mathrm{all}}$ and $K_{t}\left(s,a\right)$ for the case of QL-ES; it is thus omitted.

The two lemmas above together with (A6) imply that with probability greater than 1−2δ,

(A8) $$\begin{array}{c} \left|\Delta_{t}\right|\leq \left\{\begin{array}{cc} \gamma \sum_{i=1}^{t}{∥\Delta_{i-1}∥}_{\infty }\prod_{j=i+1}^{t}\left(I-\Lambda_{j}\right)\Lambda_{i}1+\tau_{1}{∥V^{⋆}∥}_{\infty }1+{∥\Delta_{0}∥}_{\infty }1 & t<t_{\mathrm{cover},\mathrm{all}}\\ \gamma \sum_{i=1}^{t}{∥\Delta_{i-1}∥}_{\infty }\prod_{j=i+1}^{t}\left(I-\Lambda_{j}\right)\Lambda_{i}1+\tau_{1}{∥V^{⋆}∥}_{\infty }1+{\left(1-\alpha \right)}^{t/(2t_{\mathrm{cover},\mathrm{all}})}{∥\Delta_{0}∥}_{\infty }1 & t_{\mathrm{cover},\mathrm{all}}\leq t\leq T \end{array}\right. \end{array}$$

Now, a refined recursive analysis is conducted (in Appendix B.3):

**Lemma** **A4.**
*Let*

(A9)
$$\begin{array}{cc} \; & u_{0}=\frac{{∥\Delta_{0}∥}_{\infty }}{1-\gamma },\;u_{t}={∥v_{t}∥}_{\infty } \end{array}$$

(A10)
$$\begin{array}{cc} \; & v_{t}=\left\{\begin{array}{cc} \gamma \sum_{i=1}^{t}\prod_{j=i+1}^{t}\left(I-\Lambda_{j}\right)\Lambda_{i}1u_{i-1}+{∥\Delta_{0}∥}_{\infty }1 & 1\leq t\leq t_{th}\\ \gamma \sum_{i=1}^{t}\prod_{j=i+1}^{t}\left(I-\Lambda_{j}\right)\Lambda_{i}1u_{i-1} & t>t_{th}. \end{array}\right. \end{array}$$

*Then with probability greater than 1−2δ,*

(A11)
$$\begin{array}{c} {∥\Delta_{t}∥}_{\infty }\leq \frac{\tau_{1}{∥V^{⋆}∥}_{\infty }}{1-\gamma }+u_{t}+\varepsilon \;. \end{array}$$

This lemma is the consequence of a direct computational induction from (A8), using the fact that ${\left(1-\alpha \right)}^{\frac{t}{2t_{\mathrm{cover},\mathrm{all}}}}\leq \left(1-\gamma \right)\varepsilon$ when $t\geq t_{\mathrm{th}}$, and ${∥\Delta_{0}∥}_{\infty }={∥Q^{⋆}∥}_{\infty }\leq \frac{1}{1-\gamma }$.

**Lemma** **A5.**
*Let $w_{k}={\left(1-\rho \right)}^{k}\frac{{∥\Delta_{0}∥}_{\infty }}{1-\gamma }$ for all k∈N. Then, with probability 1−2δ:*

(A12)
$$\begin{array}{c} u_{t}\leq w_{k_{t}}\;\mathrm{with}\;k_{t}=\max\left\{0,\left\lfloor \frac{t-t_{th}}{t_{cover,all}}\right\rfloor \right\} \end{array}$$

The proof for Lemma A5 relies once again on computational arguments and an induction, as showcased in Appendix B.4.

Finally, the following theorem is the last milestone of the proof, obtained from the two previous lemmas.

**Theorem** **A1.**
*For any $\varepsilon \in (0,\frac{1}{1-\gamma }]$, δ∈(0,1), there exists a universal constant κ>0 such that with probability at least 1−6δ, for all t≤T:*

(A13)
$$\begin{array}{c} ∥Q_{t}-Q^{⋆}{∥}_{\infty }\leq {\left(1-\rho \right)}^{k}\frac{∥Q_{0}-Q^{⋆}{∥}_{\infty }}{1-\gamma }+\frac{\kappa \gamma }{1-\gamma }{∥V^{⋆}∥}_{\infty }\sqrt{\alpha \log\left(\frac{CT}{\delta }\right)}+\varepsilon \end{array}$$

*with $k=\max\left\{0,\lfloor \frac{t-t_{th}}{t_{cover,all}}\rfloor \right\}$.*

It is easy to upper bound the second term in (A13) with ε by setting $\alpha =\frac{{\left(1-\gamma \right)}^{4}\varepsilon^{2}}{\kappa^{2}\gamma^{2}\log\left(CT/\delta \right)}$. Then, using ${\left(1-\rho \right)}^{k}\leq e^{-\rho k}$ and noting that $\rho \geq \frac{1}{4}\left(1-\gamma \right)\alpha$ for $\alpha <\frac{1}{2}$ allow us to upper bound the first term by ε, thanks to the definition of *k*, as long as

(A14) $$\begin{array}{cc} \; & t\geq t_{\mathrm{th}}+t_{\mathrm{cover},\mathrm{all}}+\frac{4t_{\mathrm{cover},\mathrm{all}}}{(1-\gamma )\alpha }\log\left(\frac{{∥\Delta_{0}∥}_{\infty }}{\varepsilon (1-\gamma )}\right)=T, \end{array}$$

which concludes this proof of Theorem 1.

## Appendix B. Proofs of Technical Lemmas

### Appendix B.1. Proof of Lemma A1

This proof reproduces the one of Lemma 5 in [9] but tailored to the case of QL-ES. We set $t_{l}=t_{\mathrm{cover},C}l$, and define

$$H_{l}=\left\{\exists c\in C\;\mathrm{that}\;\mathrm{is}\;\mathrm{not}\;\mathrm{visited}\;\mathrm{within}\;(t_{l},t_{l+1}]\right\}$$

for any integer l>0. From the definition of $t_{\mathrm{cover},C}$, for any $c^{\prime }\in C$,

$$\begin{array}{c} P\left(H_{l}\;|\;c_{t_{l}}=c^{\prime }\right)\leq \frac{1}{2}\;. \end{array}$$

Hence, using the Markov property, for any integer L>0,

$$\begin{array}{cc} P(H_{1}\cap & \cdots \cap H_{L})=P\left(H_{l}\cap \cdots \cap H_{L-1}\right)P\left(H_{L}\;|\;H_{1}\cap \cdots \cap H_{L-1}\right)\\ \; & =P\left(H_{1}\cap \cdots \cap H_{L-1}\right)\sum_{c^{\prime }\in C}P\left(H_{L}\;|\;c_{t_{l}}=c^{\prime }\right)P\left(c_{t_{l}}=c^{\prime }\;|\;H_{1}\cap \cdots \cap H_{L-1}\right)\\ \; & \leq \frac{1}{2}P\left(H_{1}\cap \cdots \cap H_{L-1}\right)\sum_{c^{\prime }\in C}P\left(c_{t_{l}}=c^{\prime }\;|\;H_{1}\cap \cdots \cap H_{L-1}\right)\\ \; & =\frac{1}{2}P\left(H_{1}\cap \cdots \cap H_{L-1}\right). \end{array}$$

An easy recursive derivation gives $P\left(H_{1}\cap \cdots \cap H_{L}\right)\leq 2^{-L}$. Finally:

$$P\left(\exists c\in C\;\mathrm{not}\;\mathrm{visited}\;\mathrm{between}\;(0,t_{\mathrm{cover},\mathrm{all}}]\right)\leq P\left(H_{1}\cap \cdots \cap H_{\log_{2}\frac{T}{\delta }}\right)\leq \frac{1}{2^{\log_{2}\frac{T}{\delta }}}=\frac{\delta }{T}$$

from which we easily deduce the result by using a straightforward union bound.

### Appendix B.2. Proof of Lemma A2

The proof is an adaptation of the proof of Lemma 1 in [9] to the case of Q-value updates in QL-ES. Similar to the proof of Lemma 1 in [9], we begin with looking at the (s,a)-th element of $\beta_{1,t}$:

$$\begin{array}{c} \beta_{1,t}\left(s,a\right)=\gamma \sum_{k=1}^{K_{t}\left(s,a\right)}{\left(1-\alpha \right)}^{K_{t}\left(s,a\right)-k}\alpha \left(P_{t_{k}\left(s,a\right)+1}\left(s,a\right)-P\left(s,a\right)\right)V^{⋆}\;, \end{array}$$

where $t_{k}\left(s,a\right)$ denotes the time step when c(s,a) is visited for the *k*-th time, and where $K_{t}\left(s,a\right)$ denotes the number of times (s,a) is updated during the *t* first time steps: $K_{t}\left(s,a\right)=\max\left\{k\;|\;t_{k}\left(s,a\right)\leq t\right\}$. We simplify the notation below with $t_{k}\left(s,a\right)=t_{k}$. Now, we claim that

$$\begin{array}{c} \forall \;\left(s,a\right)\in S\times A,\;\left|\beta_{1,t}\right|\leq \gamma \sqrt{\alpha \log\left(\frac{CT}{\delta }\right)}{∥V^{⋆}∥}_{\infty }\;. \end{array}$$

Firstly, the vectors $P_{t_{k}+1}\left(s,a\right),k=1,\ldots ,K$ are independent and identically distributed for any (s,a)∈S×A and any $K\in N^{⋆}$. Indeed, for any $i_{1},\cdots i_{K}\in S$,

$$\begin{array}{cc} \; & P\left(\sigma_{s,a}^{-1}\left(\sigma_{s_{t_{k}},a_{t_{k}}}\left(s_{t_{k}+1}\right)\right)=i_{k},\;\forall \;1\leq k\leq K\right)\\ & =P\left(\sigma_{s,a}^{-1}\left(\sigma_{s_{t_{k}},a_{t_{k}}}\left(s_{t_{k}+1}\right)\right)=i_{k},\;\forall \;1\leq k\leq K-1\;\mathrm{and}\;\sigma_{s,a}^{-1}\left(\sigma_{s_{t_{K}},a_{t_{K}}}\left(s_{t_{K}+1}\right)\right)=i_{K}\right)\\ \; & =\sum_{m\in N^{⋆}}P\left(\sigma_{s,a}^{-1}\left(\sigma_{s_{t_{k}},a_{t_{k}}}\left(s_{t_{k}+1}\right)\right)=i_{k},\;\forall \;1\leq k\leq K-1\;\mathrm{and}\;t_{K}=m\;\mathrm{and}\;\sigma_{s,a}^{-1}\left(\sigma_{s_{m},a_{m}}\left(s_{m+1}\right)\right)=i_{K}\right)\\ \; & \overset{\left(i\right)}{=}\sum_{m\in N^{⋆}}[P\left(\sigma_{s,a}^{-1}\left(\sigma_{s_{t_{k}},a_{t_{k}}}\left(s_{t_{k}+1}\right)\right)=i_{k},\;\forall \;1\leq k\leq K-1\;\mathrm{and}\;t_{K}=m\right)\\ & \;\times P\left(\sigma_{s,a}^{-1}\left(\sigma_{s_{m},a_{m}}\left(s_{m+1}\right)\right)=i_{K},\;|\;\left(s,a\right)\in c_{m}\right)]\\ \; & \overset{\left(ii\right)}{=}P_{s,a}\left(i_{K}\right)\sum_{m\in N^{⋆}}P\left(\sigma_{s,a}^{-1}\left(\sigma_{s_{t_{k}},a_{t_{k}}}\left(s_{t_{k}+1}\right)\right)=i_{k},\;\forall \;1\leq k\leq K-1\;\mathrm{and}\;t_{K}=m\right)\\ \; & =P_{s,a}\left(i_{K}\right)P\left(\sigma_{s,a}^{-1}\left(\sigma_{s_{t_{k}},a_{t_{k}}}\left(s_{t_{k}+1}\right)\right)=i_{k},\;\forall \;1\leq k\leq K-1\right) \end{array}$$

where (i) uses the Markov property, $\left(s_{t_{K}},a_{t_{K}}\right)\in c\left(s,a\right)$ from the definition of $t_{K}=t_{K}\left(s,a\right)$, (ii) uses the equivalence property between (s,a) and $\left(s_{m},a_{m}\right)$, and $P_{s,a}$ is the transition probability from (s,a). By induction, one obtains

$$\begin{array}{c} P\left(\sigma_{s,a}^{-1}\left(\sigma_{s_{t_{k}},a_{t_{k}}}\left(s_{t_{k}+1}\right)\right)=i_{k},\;\forall \;1\leq k\leq K\right)=\prod_{j=1}^{K}P_{s,a}\left(i_{j}\right) \end{array}$$

which proves the sought independence. Then, following identical lines as in the proof of Lemma 1 in [9] (Lemma 1), we can use Hoeffding’s inequality to bound $\beta_{1,t}$, which yields

$$\begin{array}{c} \left|\sum_{k=1}^{K}{\left(1-\alpha \right)}^{K-k}\alpha \left(P_{t_{k}+1}\left(s,a\right)-P\left(s,a\right)\right)V^{⋆}\right|\leq \sqrt{\alpha \log\left(\frac{CT}{\delta }\right)}{∥V^{⋆}∥}_{\infty }. \end{array}$$

The proof is concluded by taking the union bound over all classes c∈C and all 1≤K≤T.

### Appendix B.3. Proof for Lemma A4

The proof is a straightforward adaptation of the proof of [9] (Lemma 3), where we reproduce most of the steps from [9] for completeness. We prove the lemma by induction on *t*. The base case t=0 holds trivially:

$${∥\Delta_{0}∥}_{\infty }\leq \frac{{∥\Delta_{0}∥}_{\infty }}{1-\gamma }\leq \frac{\tau_{1}{∥V^{⋆}∥}_{\infty }}{1-\gamma }+u_{0}+\varepsilon .$$

Now let us assume that the recursive hypothesis holds for all i<t. Let us define

$$\begin{array}{c} h\left(t\right)=\left\{\begin{array}{cc} {∥\Delta_{0}∥}_{\infty } & \;\mathrm{if}\;t\leq t_{\mathrm{th}}\\ (1-\gamma )\varepsilon & \;\mathrm{if}\;t>t_{\mathrm{th}} \end{array}\right. \end{array}$$

and we remind that ${\left(1-\alpha \right)}^{\frac{t}{2t_{\mathrm{cover},\mathrm{all}}}}\leq \left(1-\gamma \right)\varepsilon$ whenever $t\geq t_{\mathrm{th}}$. Therefore, we have

$$\begin{array}{cc} \; & \left|\Delta_{t}\right|\leq \gamma \sum_{i=1}^{t}\prod_{j=i+1}^{t}\left(I-\Lambda_{j}\right)\Lambda_{i}1\left(\frac{\tau_{1}{∥V^{⋆}∥}_{\infty }}{1-\gamma }+u_{i-1}+\varepsilon \right)+\tau_{1}{∥V^{⋆}∥}_{\infty }1+h\left(t\right)1\\ \; & =\gamma \sum_{i=1}^{t}\prod_{j=i+1}^{t}\left(I-\Lambda_{j}\right)\Lambda_{i}1u_{i-1}+\gamma \sum_{i=1}^{t}\prod_{j=i+1}^{t}\left(I-\Lambda_{j}\right)\Lambda_{i}1\left(\frac{\tau_{1}{∥V^{⋆}∥}_{\infty }}{1-\gamma }+\varepsilon \right)+\tau_{1}{∥V^{⋆}∥}_{\infty }1+h\left(t\right)1. \end{array}$$

Furthermore, define the diagonal matrix $M_{i}=\prod_{j=i+1}^{t}\left(I-\Lambda_{j}\right)\Lambda_{i}$, and denote by $N_{i}^{j}\left(s,a\right)$ the number of visits to the equivalence class of the state–action pair (s,a) between the *i*-th and the *j*-th iterations (including *i* and *j*). Then the diagonal entries of $M_{i}$ satisfy

$$\begin{array}{c} M_{i}\left(\left(s,a\right),\left(s,a\right)\right)=\left\{\begin{array}{cc} \alpha {\left(1-\alpha \right)}^{N_{i+1}^{t}\left(s,a\right)},\; & \mathrm{if}\;\left(s,a\right)\in c_{i-1},\\ 0, & \mathrm{if}\;\left(s,a\right)\notin c_{i-1}. \end{array}\right. \end{array}$$

Introducing $e_{\left(s,a\right)}\in R^{SA}$ as the standard basis vector whose only non-zero entry is the (s,a)-th entry, one obtains the following:

$$\begin{array}{c} \prod_{j=i+1}^{t}\left(I-\Lambda_{j}\right)\Lambda_{i}1=M_{i}1=M_{i}e_{\left(s_{i-1},a_{i-1}\right)}=\alpha {\left(1-\alpha \right)}^{N_{i+1}^{t}\left(s_{i-1},a_{i-1}\right)}e_{\left(s_{i-1},a_{i-1}\right)} \end{array}$$

and

(A15) $$\begin{array}{cc} \sum_{i=1}^{t}\prod_{j=i+1}^{t}\left(I-\Lambda_{j}\right)\Lambda_{i}1\; & =\sum_{i=1}^{t}\alpha {\left(1-\alpha \right)}^{N_{i+1}^{t}\left(s_{i-1},a_{i-1}\right)}e_{\left(s_{i-1},a_{i-1}\right)}\\ \; & =\sum_{(s,a)\in S\times A}\left\{\sum_{i=1}^{t}\alpha {\left(1-\alpha \right)}^{N_{i+1}^{t}\left(s,a\right)}I\left\{\left(s,a\right)\in c_{i-1}\right\}\right\}e_{\left(s,a\right)}\\ \; & \leq \sum_{(s,a)\in S\times A}\sum_{j=0}^{\infty }\alpha {\left(1-\alpha \right)}^{j}e_{\left(s,a\right)}=\sum_{j=0}^{\infty }\alpha {\left(1-\alpha \right)}^{j}1=1. \end{array}$$

Using (A15), we obtain

$$\begin{array}{cc} \left|\Delta_{t}\right|\; & \leq \gamma \sum_{i=1}^{t}\prod_{j=i+1}^{t}\left(I-\Lambda_{j}\right)\Lambda_{i}1u_{i-1}+\frac{\gamma \tau_{1}{∥V^{*}∥}_{\infty }}{1-\gamma }1+\gamma \varepsilon 1+\tau_{1}{∥V^{*}∥}_{\infty }1+h\left(t\right)1\\ \; & =\frac{\tau_{1}{∥V^{*}∥}_{\infty }}{1-\gamma }1+\gamma \varepsilon 1+\gamma \sum_{i=1}^{t}\prod_{j=i+1}^{t}\left(I-\Lambda_{j}\right)\Lambda_{i}1u_{i-1}+h\left(t\right)1\\ \; & =\frac{\tau_{1}{∥V^{*}∥}_{\infty }}{1-\gamma }1+\gamma \varepsilon 1+v_{t}+\left(1-\gamma \right)\varepsilon I\left\{t>t_{\mathrm{th}}\right\}1\\ \; & \leq \frac{\tau_{1}{∥V^{*}∥}_{\infty }}{1-\gamma }1+\varepsilon 1+v_{t}\;. \end{array}$$

With the definition of $u_{t}={∥v_{t}∥}_{\infty }$, we arrive at the desired result.

### Appendix B.4. Proof for Lemma A5

This proof follows identical steps as in the proof of Lemma 4 in [9] except that the quantities $N_{i}^{n}\left(s,a\right)$ used there should be defined as the number of visits to the equivalence class of the state–action pair (s,a) between iteration *i* and iteration *n* (including *i* and *n*).
